# Karyotype and COI sequences of *Chironomus sokolovae* Istomina, Kiknadze et Siirin, 1999 (Diptera,Chironomidae) from the bay of Orkhon River, Mongolia

**DOI:** 10.3897/CompCytogen.v15i2.66549

**Published:** 2021-05-27

**Authors:** Viktor V. Bolshakov, Alexander A. Prokin

**Affiliations:** 1 Papanin Institute for Biology of Inland Waters Russian Academy of Sciences, Yaroslavl reg., Nekouz prov., Borok, 152742, Russia Papanin Institute for Biology of Inland Waters Russian Academy of Sciences Borok Russia; 2 Cherepovets State University, Lunacharski 5,Cherepovets, 162600, Vologda Oblast’, Russia Cherepovets State University Cherepovets Russia

**Keywords:** Chironomidae, *Chironomus sokolovae*, COI, Diptera, DNA-barcode, karyotype, Mongolia

## Abstract

*Chironomus sokolovae* Istomina, Kiknadze et Siirin, 1999 (Diptera, Chironomidae) is recorded from Mongolia for the first time. Eleven banding sequences determined in the Mongolian population were previously known from Altai and Yenisei populations: sokA1, sokB1, sokB2, sokC1, sokC2, sokD1, sokD2, sokE1, sokF1, sokF2 and sokG1. The additional B-chromosomes are absent. DNA-barcoding of COI gene was carried out for this species for the first time. The phylogenetic tree estimated by Bayesian inference showed a high similarity of the studied species with *Ch. acutiventris* Wülker, Ryser et Scholl, 1983 from the *Chironomus obtusidens*-group. The estimated genetic distance K2P between *Ch. sokolovae* and *Ch. acutiventris* is much lower (0.38%) than the commonly accepted threshold of 3% for species of genus *Chironomus* Meigen, 1803. Our results show that the accepted cytogenetic criteria of species level in the genus *Chironomus* are more in accordance with morphological ones of the same level, than with molecular-genetic criteria accepted for species COI genetic distance.

## Introduction

At present time, nine species of *Chironomus* Meigen, 1803 identified by imago (Hayford 2005; Shcherbina and Zelentsov 2008) are recorded from Mongolia. Seven additional species identified by imago were described from the country as new for science (Sasa and Suzuki 1997) but never found after the original description. Macrozoobenthos of the Orkhon (Kharkhorin) Reservoir on the Orkhon River near Kharkhorin city and river sections upstream and downstream of the reservoir, were studied for the first time during the fieldwork of the Joint Russian-Mongolian Complex Biological Expedition in 2017. Further research has shown that the species *Chironomus sokolovae*Istomina et al. 1999 was erroneously recorded by larvae as *Ch. obtusidens* Goethebuer, 1937 (Prokin and Sazhnev 2019) from the reservoir and connected sections of the Orkhon River with a total number of specimens up to 5000 ind/ m2 and the total biomass up to 3,75 g/m2. Larvae of *Ch. commutatus* Keyl, 1960 co-occurred with *Ch. sokolovae* (Prokin and Sazhnev 2019).

The species *Ch. sokolovae* belongs to *Chironomus obtusidens*-group including six species: *Ch. acutiventris* Wülker, Ryser et Scholl, 1983; *Chironomus bavaricus* Wülker, Ryser et Scholl, 1983; *Ch. obtusidens* Goethebuer, 1937; *Ch. arcustylus* Siirin, 2002; *Ch. heterodentatus* Konstantinov, 1956; *Ch. sokolovae* Istomina, Kiknadze et Siirin, 1999 (Siirin et al. 2002; Kiknadze et al. 2016). Chromosomal polymorphism and cytogenetic differentiation in this group are still poorly studied (Siirin et al. 2002; Kiknadze et al. 2007).

The known range of the species includes the Altai Krai, Altai Republic, and the Tyva Republic in Russia (Istomina et al. 2000; Siirin et al. 2002), and Mongolia (this publication). The species was described from the Chemal River (Altai Republic) and recorded from different water bodies in the Altai region and the Tyva Republic, where it co-occurred with *Ch. acutiventris* and *Ch. heterodentatus* (Istomina et al. 2000; Siirin et al. 2002). Numerous populations of *Ch. sokolovae* larvae inhabit silty sand of the Enisey River ripal zone, near the confluence of the Bolshoy and the Maliy Enisey Rivers (environs of Kyzyl city) (Siirin et al. 2002).

The karyotype and DNA barcoding of COI gene of the *Ch. sokolovae* from the Orkhon River (Mongolia) are described in this publication with the aim of clarifying the species position within the *Ch. obtusidens*-group.

## Materials and methods

Two larvae were collected from the bay of the Orkhon River upstream of the reservoir: 47°10.734'N,102°47.384'E, in September 2017. Depth 0.5 m, bottom – silty sand. Temperature 20.0 °C, pH 7.2, EC 172 mkSm/sm, TDS = 98 mg/l. For all analyses larvae were fixed in ethanol (95%). Two fourth instar larvae were used for karyotype analysis by the ethanol-orcein technique (Dyomin 1989). A Micromed-6C (LOMO, St. Petersburg) light microscope equipped with standart (kit) oil objective ×100, and camera ToupCam5.1 (China) were used for microscopy analysis. Cytomaps from Kiknadze et al. (2016), Keyl (1962), Wülker et al. (1983) and Dévai et al. (1989) were used to identify chromosome banding.

One larva which was studied karyologically was taken for the total DNA extraction using «M-sorb-OOM» (Sintol, Moscow) kit with magnet particles according to manufacturer’s protocol. For amplification of COI (cytochrome oxidase subunit I) we used primers LCO1490 (5’-GGTCAACAAATCATAAAGATATTGG-3’) and HCO2198 (5’-TAAACTTCAGGGTGACCAAAAAATCA -3’) (Evrogen, Moscow) (Folmer et al. 1994). Amplification reaction was carried out in 25 μLreaction mixture (1× buffer, 1.5 μM MgCl2, 0.5 mM of each primer, 0.2 μM dNTP of each nucleotide, 17.55 μL deionized water, 1 μL template DNA, 1 unit Taq-polymerase (Evrogen, Moscow). PCR performed at 94 °C (3 min), followed by 30 cycles at 94 °C (15 s), 50 °C (45 s), 72 °C (60 s) and a final extention at 72 °C (8 min). PCR products were visualized on 1% agarose gels and later purified by ethanol and ammonium acetate (3 M). Both strands were sequenced on an Applied Biosystems 3500 DNA sequencer (Thermo Scientific, USA) following the manufacturer’s instructions.

For alignment of COI nucleotide sequences we used MUSCLE in the MEGA6 software (Tamura et al. 2013). The MEGA6 was used to calculate pairwise genetic distances Kimura 2-parameter (K2P) with codon position preferences: 1^st^, 2^nd^, 3^rd^ and noncoding sites (Kimura 1980). The Bayesian analysis was performed using the program MrBayes v.3.2.6 (Ronquist and Huelsenbeck 2003; Ronquist et al. 2012) with settings suggested by Karmokov (2019), for 1,000,000 iterations and 1000 iterations of burn-in, nst = 6 (GTP + I + G). The phylogenetic trees resulting in Bayesian inference analyses were visualized and edited using FigTree v.1.4.3 (http://tree.bio.ed.ac.uk/software/figtree/).

In addition, the 27 COI sequences of the genus *Chironomus* from “GenBank” and “Barcode of Life Data Systems” (BOLD; http://www.boldsystems.org) were analyzed. Accession numbers of used sequences in GenBank and BOLD: *Chironomus acutiventris* Wülker, Ryser et Scholl 1983 (AF192200.1), *Ch. annularius* Meigen, 1818 (AF192189.1), *Ch. aprilinus* Meigen, 1830 (KC250746.1), *Ch. balatonicus* Devai, Wulker et Scholl, 1983 (JN016826.1), *Ch. bernensis* Wülker & Klötzli, 1973 (AF192188.1), *Ch. borokensis* Kerkis, Filippova, Schobanov, Gunderina et Kiknadze, 1988 (AB740261), *Ch. cingulatus* Meigen, 1830 (AF192191.1), *Ch. commutatus* Keyl, 1960 (AF192187.1), *Ch. curabilis* Belyanina, Sigareva et Loginova, 1990 (JN016810.1), *Ch. dilutus* Shobanov, Kiknadze et Butler, 1999 (KF278335.1), *Ch. entis* Shobanov, 1989 (KM571024.1), *Ch. heterodentatus* Konstantinov, 1956 (AF192199.1), *Ch. heteropilicornis* Wülker, 1996 (MK795772.1), *Ch. luridus* Strenzke, 1959 (AF192203.1), *Ch. maturus* Johannsen, 1908 (DQ648204.1), *Ch. melanescens* Keyl, 1961 (MG145351.1), *Ch. nipponensis* Tokunaga, 1940 (LC096172.1), *Ch. novosibiricus* Kiknadze, Siirin & Kerkis, 1993 (AF192197.1), *Ch. nuditarsis* Keyl, 1961 (KY225345.1), *Ch. obtusidens* Goetghebuer, 1921 (CHMNO207-15*); *Ch. piger* Strenzke, 1959 (AF192202.1), *Ch. pilicornis* Fabricius, 1787 (BSCHI736-17*), *Ch. plumosus* (Linnaeus, 1758) (KF278217.1), *Ch. riparius* Meigen, 1804 (KR756187.1), *Ch. tentans* Fabricius, 1805 (AF110157.1), *Ch. tuvanicus* Kiknadze, Siirin et Wülker, 1993 (AF192196.1), *Ch. whitseli* Sublette & Sublette, 1974 (KR683438.1). The COI sequence of *Ptychoptera minuta* Tonnoir, 1919 (KF297888) was used as outgroup in phylogenetic analysis.

## Results and discussions

### Karyotype of *Ch. sokolovae* from the Orkhon River, Mongolia

The chromosome set of the species is 2n = 8. The cromosome arm combination is AB, CD, EF and G (the *Chironomus* “thummi” cytocomlex). The additional B-chromosomes are absent. The chromosomes AB and CD are metacentric, EF is submetacentric, and G is telocentric. The karyotype of *Ch. sokolovae* is similar to *Ch. acutiventris*, but differs by fixed inversions in arms B, C, D and F (Kiknadze et al. 2016).

**Figure 1.**
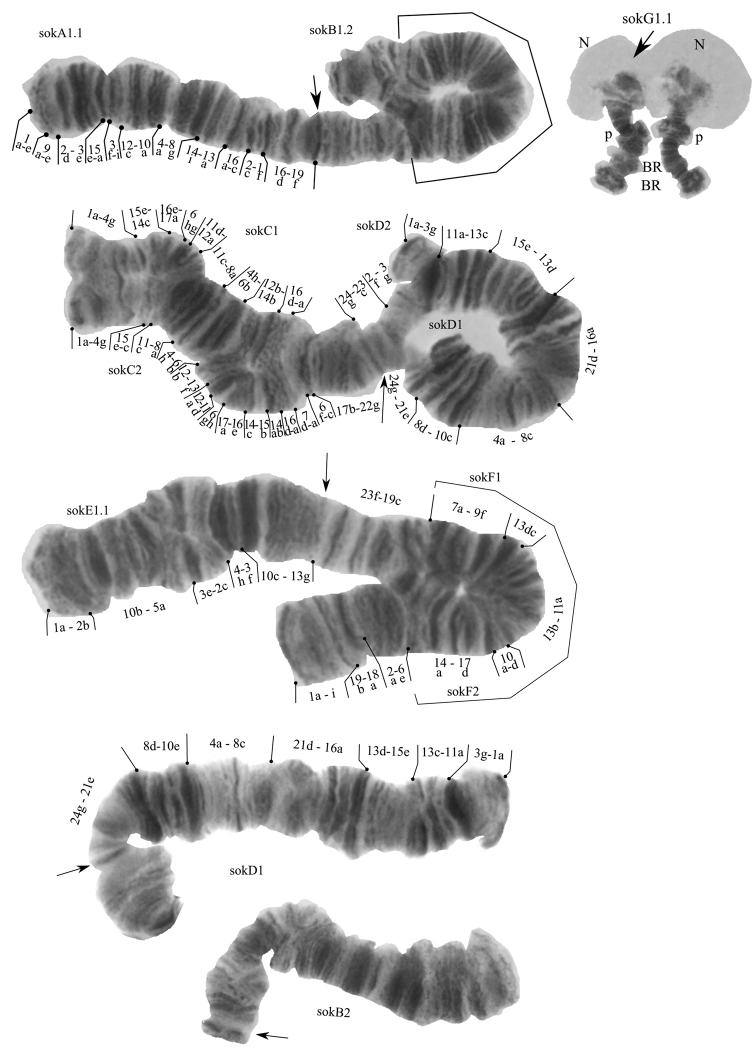
Banding sequences of *Ch. sokolovae* from the Orkhon River, Mongolia. Arrows indicate centromeric band, sokA1, sokB1 and etc. – genotypic combinations of banding sequences in chromosome arms, Br – Balbiani rings, N – nucleous, p – puffs. sokA1.1. – mapped according to picture 2.51.2 by Kiknadze et al. (2016).

We found two different karyotypes (genotypic combinations) in both larvae from Mongolia: sokA1.1.B1.1.C1.2.D1.1.E1.1.F1.1.G1.1 and sokA1.1.B1.2.C1.2.D1.2.E1.1.F1.2.G1.1., which consist of 11 banding sequences out of 18 known for the karyofund of this species (Istomina et al. 1999; Siirin et al. 2002) (Fig. 1).

**Arm A.** One banding sequence sokA1 1a-e 9a-e 2d-3e 15e-a 3f-i 12c-10a 4a-8g 14i-13a 16a-c 2c-1f 16d-19f C.

**Arm B.** Two banding sequences: sokB2 was found in homozygous and heterozygous state with sokB1. Both banding sequences are still not mapped.

**Arm C.** Two banding sequences: sokC1 1a-4g 15e-14c 16e-17a 6hg 11d-12a 11c-8a 4h-6b 12b-14b 16d-a 7d-a 6f-c 17b-22g C, found in heterozygous state with sokC2 1a-4g 15e-c 11c-8a 4h-6b 12b-13f 12a-11d 6gh 17a-16e 14c-15b 14ab 16d-a 7d-a 6f-c 17b-22g C.

**Arm D.** Two banding sequences: sokD1 1a-2e 23b-21e 8d-10e 4a-8c 21d-16a 13d-15e 13c-11a 3g-2f 23c-24g C found in homozygous and heterozygous state with sokD2 1a-3g 11a-13c 15e-13d 16a-21d 8c-4a 10e-8d 21e-24g C.

**Arm E.** One banding sequence sokE1 1a-2b 10b-5a 3e-2c 4h-3f 10c-13g C.

**Arm F.** Two banding sequences: sokF1 1a-i 19b-18a 2a-9f 13dc 11a-13b 10d-a 17d-14a 19c-23f C found in homozygous and heterozygous state with sokF2 1a-i 19b-18a 2a-6e 14a-17d 10a-d 13b-11a 13cd 9f-7a 19c-23f C.

**Arm G.** One banding sequence sokG1 was found. Not mapped.

All 11 banding sequences found in Mongolian larvae were previously known for both the Enisey and the Altai populations of studied species (Istomina et al. 1999; Siirin et al. 2002). In Mongolian and Enisey populations sokB1 banding sequence was found only in the heterozygous state, whereas in the Altai population it was in the homozygous state (Istomina et al. 1999; Siirin et al. 2002).

### DNA-barcoding and phylogenetic analysis

Single nucleotide sequence of *Ch. sokolovae* for the F6.2 gene from the tissue-specific Balbiani rings locus (Alieva et al. 2004) is accessible in GenBank (AF521040), while there is no sequences for barcoding. We obtained the COI barcode for *Ch. sokolovae* with the length of 665 nucleotides (percentage A: 16.9; T: 25.3; G: 11.8; C: 12.5) and deposited it into the GenBank database with accession number – MW471100.

Pairwise genetic distances between *Ch. sokolovae* and the members of the *Ch. obtusidens* group obtained by K2P model (Kimura 1980) shown high variability. Calculated distance between sequences of *Ch. sokolovae* and *Ch. acutiventris* sequences was 0.38%, *Ch. heterodentatus* – 4.60%, *Ch. obtusidens* – 11.56%.

According to Polukonova et al. (2009) and Proulx et al. (2013) *Chironomus* COI interspecific sequence divergences is about 3%. In our study, interspecific divergence between *Ch. sokolovae* and *Ch. acutiventris* is 0.38%, that is much lower than the 3% accepted interspecific threshold. In most cases such low values of distances between species occur due to incorrect species identification when only morphological traits were used. To exclude a possibility of such mistake, we used karyological analysis and confirmed the accuracy of our species identification (Fig. 1).

**Figure 2.**
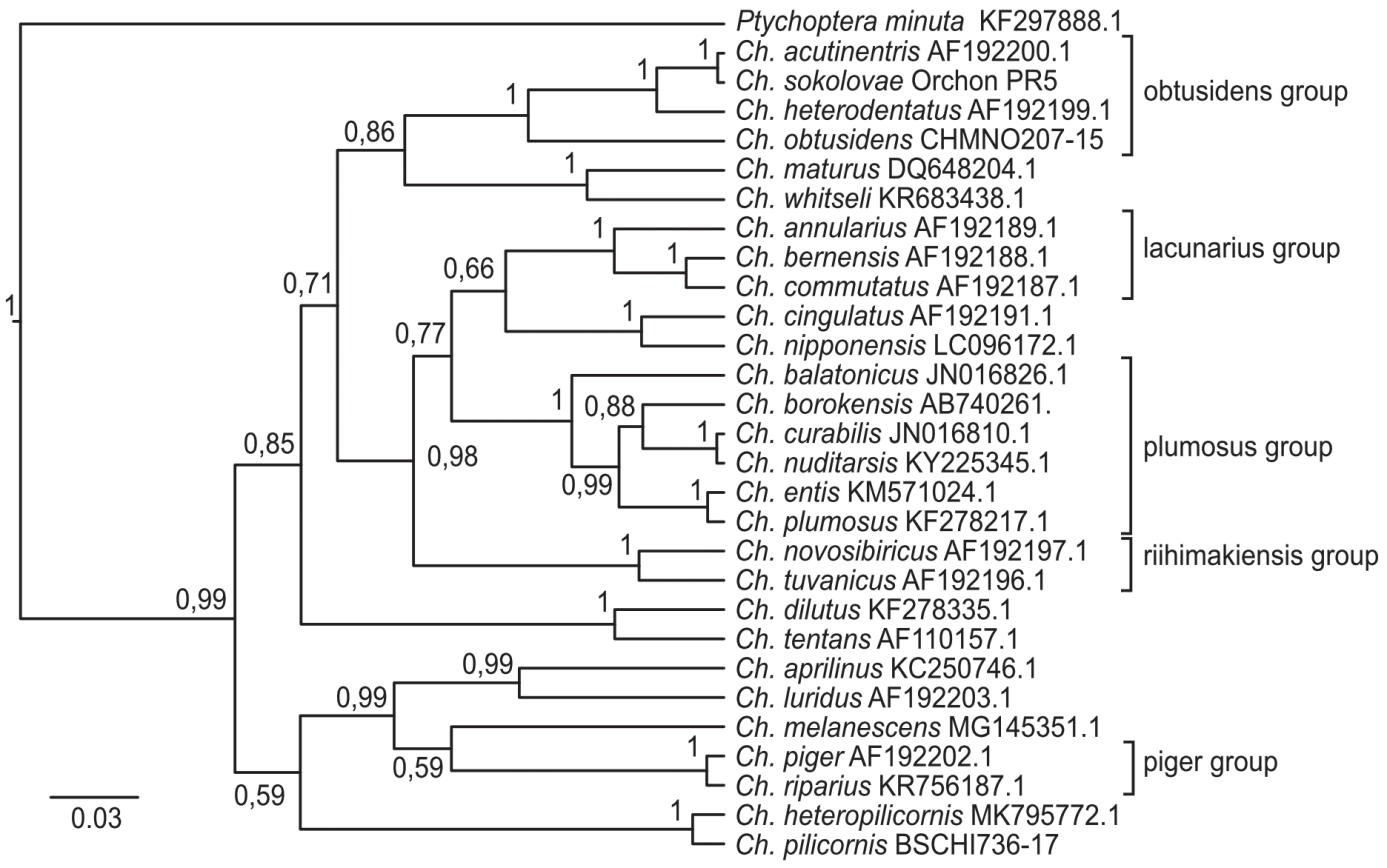
Bayesian tree of the analyzed samples of *Chironomus* spp. inferred from COI sequences. Species name, GenBank accession numbers and group name are shown to the right of the branches. Support values are given if they exceed 0.5. The numbers at the nodes indicate posterior probabilities.

Phylogenetic analysis using COI sequences showed groups of related species (Fig. 2), which concur with how these species were combined into the groups earlier on the basis of karyological and morphological traits (Kiknadze et al. 2016; Karmokov 2019). Obtained data are highly accurate (~100%) and show that *Ch. sokolovae* belongs to the *Chironomus obtusidens* group and is closest to *Ch. acutiventris*.

## Conclusions

The Species *Ch. sokolovae* and *Ch. acutiventris* are similar in their karyotypes but differ in a few fixed inversions in arms B, C, D and F (Kiknadze et al. 2016). The COI sequences of these species are also similar, which could be the effect of a close relationship between the species, indicative of their recent origin (Michailova et al. 2021), or could be the result of interspecific hybridization with fixation of mtDNA in one of the parental species in the population (Guryev and Blinov 2002; Polukonova and Dyomin 2010, 2013). Study by Siirin et al. (2002) mentioned the existence of interspecific hybrids of *Ch. sokolovae* and *Ch. heterodentatus*, which means that hybridization between the *Ch. sokolovae* and *Ch. acutiventris* still occur as a result of living together in the same habitats and co-swarming. At the same time, the mtDNA sequences mostly allows the delimitation between sibling species in such groups of species as *Ch. obtusidens*, *Ch. lacunarius*, *Ch. plumosus* etc. (Fig. 2), that was originally founded based on morphological and cytogenetics traits. For a more detailed analysis of the species position within the group is needed to perform sequencing of mitochondrial and nuclear genomes, coupled with the preliminary cytogenetic and morphological analysis as obligatory.
